# 2322. Effectiveness of Recombinant Influenza Vaccine vs. Standard Dose Inactivated Influenza Vaccines Against Hospitalized Influenza-Related Outcomes in Adults: A Cluster Randomized Trial

**DOI:** 10.1093/ofid/ofac492.153

**Published:** 2022-12-15

**Authors:** Amber Hsiao, Arnold Yee, Bruce Fireman, John R Hansen, Ned Lewis, Nicola P Klein

**Affiliations:** Division of Research Kaiser Permanente Vaccine Study Center, Oakland, California; Division of Research Kaiser Permanente Vaccine Study Center, Oakland, California; Division of Research Kaiser Permanente Vaccine Study Center, Oakland, California; Division of Research Kaiser Permanente Vaccine Study Center, Oakland, California; Division of Research Kaiser Permanente Vaccine Study Center, Oakland, California; Division of Research Kaiser Permanente Vaccine Study Center, Oakland, California

## Abstract

**Background:**

More effective influenza vaccines are needed to prevent influenza and its complications. Recombinant influenza vaccines may offer better protection than traditional egg-based vaccines. Using a cluster randomized design to estimate the relative vaccine effectiveness (rVE) of Flublok Quadrivalent recombinant influenza vaccine (RIV4) vs. standard dose inactivated influenza vaccine (SD-IIV4), we separately reported that RIV4 provided significantly better protection against primary outcome of confirmed influenza in adults 50–64 yrs in the 2018–20 moderately severe influenza seasons. Here we report the rVE against the secondary and exploratory outcomes of hospitalized influenza-related outcomes.

**Methods:**

We randomized 66 facilities within Kaiser Permanente Northern California (KPNC) to routinely administer RIV4 or SD-IIV4 to adults, alternating vaccine formulations weekly by facility during the influenza season (ClinicalTrials.gov NCT03694392). Hospitalized cases of PCR-confirmed influenza, community-acquired pneumonia, and cardio-respiratory events were prespecified as secondary in 50–64 yrs and exploratory in 18–49 yrs. We estimated the relative hazard ratio (rHR) using Cox regression on a calendar timeline, adjusting for age, sex, and race, using facility-specific stabilized propensity scores. We calculated rVE as (1–rHR) x 100%.

**Results:**

During the 2018–19 and 2019–20 seasons, the study consisted of 1,630,328 vaccinees 18–64 yrs (RIV4=632,962; SD-IIV4=997,366), and included 95 hospitalized influenza cases after RIV4 and 153 after SD-IIV4 among 50–64 yrs (Table 1). RIV4 vs. SD-IIV4 was not significantly more protective against hospitalized influenza-related outcomes in adults 50–64 yrs or 18–49 yrs.

**Conclusion:**

While this large cluster randomized study found that RIV4 vs. SD-IIV4 reduced the risk of lab-confirmed influenza in adults 50–64 yrs across 2 influenza seasons, RIV4 and SD-IIV4 performed similarly against hospitalized influenza-related outcomes. Evaluating influenza-related hospitalizations during influenza seasons when more hospital outcomes occur may help to better understand the relative effectiveness of recombinant versus standard inactivated influenza vaccines against severe disease.

**Disclosures:**

**All Authors**: No reported disclosures.

